# Frequency of Minor Skin and Soft Tissue Complications in Facial and Periorbital Area after Septorhinoplasty

**Published:** 2012

**Authors:** Shadman Nemati, Javad Golchay, Arsalan Alizadeh

**Affiliations:** 1 *Otolaryngology, head and neck surgery department and research center, Amiralmomenin Hospital, Guilan University of Medical Sciences, Rasht, Iran*; 2 *Department of dermatology, Guilan University of Medical Sciences, Rasht, Iran *; 3 *Otolaryngology, head and neck surgery research center, Amiralmomenin Hospital, Rasht, Iran*

**Keywords:** Acne, Complication, Exacerbation, Paresthesia, Rhinoplasty, Skin, Soft tissue

## Abstract

**Introduction:**

High prevalence of rhinoplasty in the community and lack of structured studies about its minor skin and soft tissue complications, point out the necessity of performing precise and comprehensive studies. The aim of this study was to determine the frequency of minor cutaneous and soft tissue complications of rhinoplasty.

**Materials and Methods:**

110 patients (30 Male and 80 Female, Mean age: 26.3± 6.8) participated in this study. Before surgery all of them were checked for having each of intended complications, and 1 and 3 months after the surgery, they underwent serial visits for monitoring skin & soft tissue complications. The software used for data analysis was SPSS ver.16.

**Results:**

Acne exacerbation was seen in 27% of cases in first post-surgical visit. Nasal tip paresthesia was the most frequent complication in both postsurgical visits (49.6% in first and 36.3% in second visit), followed by eyebrow loss (31.8%), complaint of increased yawning (31.8%), periorbital hyperpigmentation (21.8%) in first visit, and, hyperpigmentation (19.1%), complaint of increased yawning (10%) and surgical site scar (7.2%) in second visit respectively. The frequency of complications was highest in younger than 25 year old age group.

**Conclusion:**

Having knowledge about these complications helps us to know which of them needs prompt medical intervention and which of them will resolve with time and just needs giving reassurance to the patient.

## Introduction

Rhinoplasty is one of the most frequently performed cosmetic surgical procedures, has been one of the most interesting fields among plastic surgeons for many years (1). Many patients undergo this operation in order to look and function better. According to the American Society of Aesthetic Plastic Surgery, rhinoplasty was among top five common aesthetic surgical procedures in the United States and it was the most frequently performed surgical procedure for teenagers in 2009 (2). However, similar to all surgical procedures, complications may occur after rhinoplasty, the rate for major or noticeable complications is reported to be 8 –15% that may be classified as hemorrhagic, infectious, traumatic, functional, and esthetic (3,4). While majority of these do not pose as a life-threatening condition, the rarely, major complications that would threaten life such as rhinorrhea, pneumothorax, and subarachnoid hemorrhage are encountered (5-10). Usually, rhinoplasty techniques focus on alteration of the structural framework of the nose. However, the aesthetic outcome, is the product of the nasal skeleton contour and the overlying skin-soft tissue envelope (11). In our experience, we faced several minor skin and soft tissue complications like acne exacerbation, nasal tip paresthesia, periorbital hyperpigmentation etc. That had made the patients concerned about their surgical result. As we found no structured studies in this regard, we decided to perform a study that’s aim was to determine the frequency of minor skin and soft tissue complications in facial and periorbital area after rhinoplasty and septorhinoplasty.

## Materials and Methods

A descriptive longitudinal study was performed on 110 patients (80 females and 30 males) admitted for septorhinoplasty in a tertiary referral university hospital from October 2009 to June 2010. Patients who had any of these conditions were excluded: any cutaneous disease like atopic dermatitis and contact dermatitis prior to surgery; systemic diseases e.g. SLE.; prior telangiectasia on face; Prior septorhinoplasty and, any cutaneous disease needing treatment. data gathering method was direct observation and complications were as follows: 

- Nasal tip paresthesia: Patient were said to close his eyes. 

Then asked if he could sense the irritation made by a piece of paper on the tip of his nose.

-Acne exacerbation: The severity of acne was measured before the surgery and one month and three months after that by Global Acne Grading System (GAGS) which is a scoring system based on type and the number of acne lesions on face, chest and upper back areas.

- Eyebrow loss: Patients asked if there were noticeable eyebrow loss or in an extent that attract the attention of other members of the family.

- Periorbital hyperpigmentation: This complication determined as an increase in the pigmentation of periorbital skin. It’s worth mentioning that the periorbital hyperpigmentation is different from periorbital ecchymosis and patients were informed about their difference. 

- Surgical site scars: presence of any form of scars on surgical incision sites where checked by the examiner.

- Nasal incontinency: loss of nasal mucosal sensation leads to this complication. 

Patients were asked about not being aware of any nasal discharges until it gets out of the nostrils. 

- Sensation of foreign body or scar inside the nose: patients were asked about having the sensation of any foreign material or accessory tissue inside their nose. 

Any positive answer considered true just after excluding any probability of foreign body presence inside the nasal cavity by direct observation.

- Complaint of increased yawning: patients

were asked if there were any inconvenience resulted by increased yawning frequency that had also been noticed by their family members.

- Dermatitis: presence of dermatitis where checked by the dermatologist.

- Skin atrophy: which is thinning of one of the top two layers of skin, the dermis or epidermis, causing a depression in the skin,were checked by dermatologist.

- Telangiectasias: these are small superficial vessels of the skin visible to the human eye and usually measure 0.1 to 1.0 mm in diameter. The presence of this complication was checked by dermatologist.

- Red spots on nasal skin: These are tiny red macules visible by naked eye the presence of which were checked by dermatologist.

Before surgery all of the patients were checked for having each of intended complications. After initial examination, the operation was performed by a single surgeon with a constant method in similar environmental conditions under general anesthesia. Patients spent the night at the hospital and were discharged the morning after the surgery. All of the patients had nasal cast for 6 days. They had adhesive tape for 3 to 4 weeks after removing the cast. The adhesive tape covered their nose to the nasofacial sulcus. 

Patients were allowed to wash their face 5 days after the cast removal. They were visited one month and three months after the surgery in order to monitor any possible complication. 

There were two post-surgical visits with a 1 and 3 month interval between them and the surgery to facilitate covering all possible outcomes. 

The proposal of the research was reviewed and approved by the Guilan University of Medical Sciences Research Office Review Board and Ethics Committee. Statistical analysis was performed by use of SPSS version 16 software (SPSS, Inc., Chicago, IL).The level of significance was determined to be 0.05.

## Results

From 135 patients who entered the study, 110 patients (%81) including 30 male and 80 female attended both the first and second post-surgical visits. 

Hence the other patients who their follow up data was incomplete were omitted from the study.

The mean age of the patients was 26.34 6.82 years. Nasal tip paresthesia was the most frequent complication in first and second post-surgical visit followed by acne exacerbation, eyebrow loss, periorbital hyperpigmentation, surgical site scar, nasal incontinency, having the sensation of foreign body presence or scar inside the nose and dermatitis respectively in first post-surgical visit. In second post-surgical visit the other complications less frequent than nasal tip paresthesia were periorbital hyperpigmentation, surgical site scar, nasal incontinency, sensation of scar inside the nose and eyebrow loss respectively. In first visit, one patient developed dermatitis in the area covered by the adhesive tape that resolved in second visit. 

In comparison between pre-surgical and first post-surgical visit, the severity of acne increased significantly. 

In first post-surgical visit 42.9% of those who had no acne before surgery, developed mild acne and 14.5% of those who had mild acne, turned into moderate form (P= 0.0001). The prevalence of acne exacerbation was 27% in first post-surgical visit.

Patients had been divided in three age groups. Those who were, under 25 year old, 25-35 year old and over 35 year old. In both post-surgical visits and in all age groups the most frequent complication was nasal tip paresthesia. The frequency of complications was the most in under 25 year old age group. 

**Fig1:**
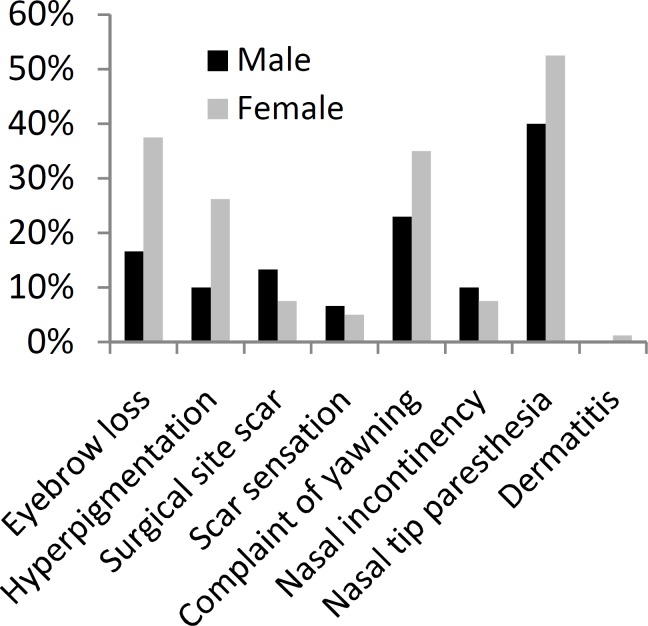
Frequency of Complications in first post-surgical visit based on gender

**Fig 2:**
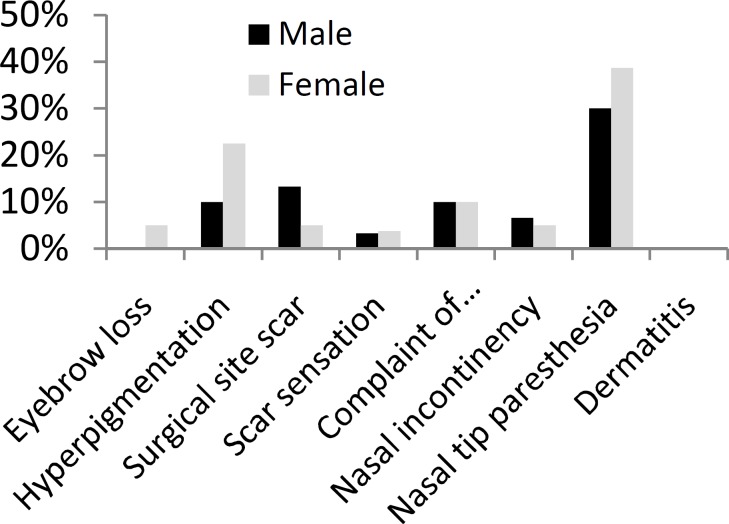
Frequency of Complications in Second post-surgical visit based on gender

Additional Information regarding the frequency of complications in first and second post-surgical visits are provided in (Tables and figures 1,2).

**Table 1:** Demographic specifications of participants

Variable		Number	Percent
Gender	Male	30	27.3
	Female	80	72.7
Age group (years)	=< 25	64	58.2
	25-35	32	29.1
	>35	14	12.7
Age (years)	Mean Variance = 26.34 6.82		

**Table 2:** Frequency of complications in post-surgical visits based on gender

Complication	First Visit				Second Visit			
	Male		Female		Male		Female	
	#	%	#	%	#	%	#	%
Eyebrow loss	5	16.6	30	37.5	0	0	4	5
Hyperpigmentation	3	10	21	26.2	3	10	18	22.5
Surgical site scar	4	13.3	6	7.5	4	13.3	4	5
Scar sensation	2	6.6	4	5	1	3.3	3	3.75
Complaint of yawning	7	23.3	28	35	3	10	8	10
Nasal incontinency	3	10	6	7.5	2	6.6	4	5
Nasal tip paresthesia	12	40	42	52.5	9	30	31	38.75
Dermatitis	0	0	1	1.2	0	0	0	0

## Discussion

Rhinoplasty is one of the most commonly performed aesthetic plastic surgeries (2). Usually, rhinoplasty techniques focus on alteration of the osteocartilagenous framework of the nasal skeleton. However, the aesthetic outcome is the product of the nasal skeleton contour and the overlying skin-soft tissue envelope. The quality of the skin is an essential indicator of the surgical outcome and plays a significant role in preoperative planning. This critical component of rhinoplasty is often underappreciated (11,12). Cutaneous problems after rhinoplasty has always been considered minor, and transient (13), Most common being contact dermatitis, appearing as rashes or pustules with or without allergic reactions from the adhesive tape (14,15). Rajabian et al. in 2004 reported a case of severe facial dermatitis in a young female one month after rhinoplasty (15). 

Cochran and Landecker in their review article in 2008 mentioned some of the cutaneous complications of rhinoplasty such as nasal cysts, contact dermatitis and skin necrosis (4). In our experience we had faced few complications in nasal and periorbital skin such as eyebrow loss, internal nasal scars and a kind of feeling about their presence by the patient, periorbital hyperpigmentation etc. that are not mentioned, even briefly, in references and various databases. Perhaps due to the presence of major structural complications and unsatisfaction from the appearance of the nose, these kind of complications which considered minor, has often been ignored by patients and surgeons. As we searched through various databases we realized there is an unignorable lack of structured studies about minor skin and soft tissue complications of rhinoplasty.

Among all 12 complications we mentioned in this study, nasal tip paresthesia was the most frequent in both post-surgical visits, which could be resulted from traumatization of sensory nerves of this area, followed by eyebrow loss and complaint of increased yawning (perhaps due to changes in nasal valve’s statics and dynamics) respectively. These two latter complications decreased with time and their frequency decreased in second post-surgical visit. Eyebrow loss is probably related to the stress caused by the surgery itself but it’s definite cause is still unknown (16). Another common complication is periorbital hyperpigmentation that frequency had decreased with time from %21.8 in first post-surgical visit to %19 in the second. Nasal incontinency and feeling of scar inside the nasal cavity were all of those complications that their frequency had increased in second post surgical visit in comparison with the first one. This increase probably happened because with time passing, most of the major concerns of the patients, like the pain caused by surgery and swelling were mostly resolved, casting and tapes were removed and these minor complications attracted the patients’ attention. 

In our study we found no incidence of skin atrophy, telangiectasia or red spots in the patients’ facial and periorbital skin. There was just one incidence of dermatitis in nasal and facial skin areas that were in contact with adhesive tape that was completely resolved in second post surgical visit after giving up using adhesive tapes. This issue suggests that the incidence of dermatitis was probably caused by sensitivity to adhesive tape. 

We analyzed more frequent complications like eyebrow loss, nasal tip paresthesia, complaint of increased yawning and hyperpigmentation according to the gender of the patients that were affected, and we found that just in case of eyebrow loss and only in first post-surgical visit compared to pre-surgical one, there was a significant difference between the frequency of this complication among males and females and females were more probable to have eyebrow loss after rhinoplasty (P<0.05). 

The frequency of all 12 complications were higher in under 25 year old age group. Because most of the admitted patients for rhinoplasty were in this age category, it’s obvious that the cumulative frequency of complications would be higher in this age group.

Due to high frequency of minor post rhinoplasty complications, lack of enough structured studies in this regard and ambiguity of the exact pathophysiological aspects and risk factors of these complications, more studies about these complications and their aggravating and resolving factors are needed. It seems that the most important issue is gaining more knowledge about the natural history of these complications.

## Conclusion

Having knowledge about minor complications of rhinoplasty will help us to know which of these complications needs prompt medical intervention and which of them will resolve with time and just needs giving reassurance to the patient.
